# *Spirulina* extract improves age-induced vascular dysfunction

**DOI:** 10.1080/13880209.2022.2047209

**Published:** 2022-03-16

**Authors:** Michal Majewski, Mercedes Klett-Mingo, Carlos M. Verdasco-Martín, Cristina Otero, Mercedes Ferrer

**Affiliations:** a Departamento de Fisiología, Facultad de Medicina, Universidad Autónoma de Madrid, Madrid, Spain; b Department of Pharmacology and Toxicology, Faculty of Medicine, University of Warmia and Mazury, Olsztyn, Poland; c Departamento de Biocatálisis, Instituto de Catálisis y Petroleoquímica, Consejo Superior de Investigaciones Científicas, Madrid, Spain; d Instituto de Investigación Hospital Universitario La Paz (IdiPAZ) Madrid, Madrid, Spain

**Keywords:** Ageing, antioxidant, nitric oxide, rat aorta, reactive oxygen species

## Abstract

**Context:**

Vascular dysfunction is considered a hallmark of ageing that has been associated with altered vasomotor responses, in which nitric oxide (NO) and reactive oxygen species participate. The consumption of *Spirulina* extracts, with antioxidant properties, increased recently.

**Objective:**

This study investigates the effect of *Spirulina* aqueous extract (SAE) on the vascular function of the aorta from aged rats.

**Materials and methods:**

Aortic segments from aged male Sprague-Dawley rats (20–22 months old) were exposed to SAE (0.1% w/v, for 3 h) to analyse: (i) the vasodilator response induced by acetylcholine (ACh), by the NO donor sodium nitroprusside (SNP), by the carbon monoxide releasing molecule (CORM) and by the K_ATP_ channel opener, cromakalim (CK); (ii) the vasoconstrictor response induced by KCl and noradrenaline (NA); (iii) the production of NO and superoxide anion, and (iv) the expression of the p-eNOS and HO-1 proteins.

**Results:**

Incubation with SAE increased the expression of p-eNOS (1.6-fold) and HO-1 (2.0-fold), enhanced NO release (1.4-fold in basal and 1.9-fold in ACh-stimulated conditions) while decreased the production of superoxide (0.7-fold). SAE also increased the sensitivity (measured as pEC_50_) to ACh (control: −7.06 ± 0.11; SAE: −8.16 ± 0.21), SNP (control: −7.96 ± 0.16; SAE: −9.11 ± 0.14) and CK (control: −7.05 ± 0.39; SAE: −8.29 ± 0.53), and potentiated the response to KCl (1.3-fold) and to NA (1.7-fold).

**Conclusion:**

The antioxidant properties of SAE improved the vasomotor responses of aorta from aged rats. These results may support the use of *Spirulina* as a protection against vascular dysfunction.

## Introduction

Ageing is a physiological process associated with vascular dysfunction that includes altered release and function of endothelial factors and vascular remodelling (Brandes et al. 2005; Thijssen et al. 2016; Ungvari et al. 2018). The crucial role of endothelial nitric oxide (NO) in vasomotor tone regulation is widely recognised. One of the major downstream events after NO release is an increase in cyclic guanosine monophosphate (cGMP) formation through soluble guanylate cyclase (sGC) stimulation, and the subsequent activation of cGMP-dependent protein kinase (cGK; Lincoln et al. 2001). On the other hand, NO bioavailability is determined by the rate of NO production and by its scavenging by superoxide anion, and several studies have shown increased vascular formation of superoxide anion with ageing (Wu et al. 2014; Daiber et al. 2017).

Under pathophysiological conditions associated with increased oxidative stress, different homeostatic mechanisms can be activated. Thus, nuclear factor erythroid 2-related factor 2 (Nrf2) mediates the transcription of phase II antioxidant proteins responsible for the elimination of reactive oxygen species (ROS; Howden 2013) and hemeoxygenase-1 (HO-1). This process has been recognised as one of the most important factors protecting vascular tissue from a pro-oxidant environment (Kang et al. 2015; Drummond et al. 2019). This enzyme synthesises carbon monoxide (CO) from the degradation of haem to biliverdin/bilirubin. This gas mediator molecule is able to activate GC, increase cGMP levels and induce relaxation (Ryter et al. 2006). In addition, both NO (Félétou and Vanhoutte, 1996) and CO (Leffler et al. 2006) are able to activate potassium channels and induce membrane hyperpolarization in vascular smooth muscle cells. Thus, the participation of hyperpolarizing mechanisms through the activation of calcium- and ATP-dependent potassium channels (K_Ca_ or K_ATP_, respectively), in conditions with decreased NO bioavailability, has been demonstrated in conductance and resistance vessels (Edwards et al. 2010; Dogan et al. 2019).

Since the development of cardiovascular diseases (CVD) are related to increased oxidative stress (Daiber et al. 2017; Dubois-Deruy et al. 2020), antioxidant therapies have been proposed to prevent endothelial and vascular dysfunction (Mozaffarian and Wu 2011; Villalpando et al. 2015; Rojas et al. 2020). Also, the consumption of natural products is gaining attention, especially that of microalgae. Among the microalgae, the filamentous cyanobacterium of the genus *Arthrospira*, with the two most common species (*Arthrospira platensis* and *Arthrospira maxima*), commercially named as *Spirulina*, is referred to as a ‘superfood’ with antioxidant-antihypertensive activities, insulin resistance and cholesterol/lipid-lowering effects (Hosseini et al. 2013; Vo and Kim 2013; Heo et al. 2017).

The consideration of these data led us to hypothesise that *Spirulina* aqueous extract could ameliorate the ageing-related vascular dysfunction. Therefore, the present study analyzes the effect of *Spirulina* aqueous extract on the vasomotor function of isolated aorta from aged rats, as well as the modulatory effects underlying superoxide anion production and NO bioavailability.

## Materials and methods

### Drugs and reagents

The drugs used were hydroethidine (HE), L-noradrenaline (NA) hydrochloride, ACh chloride, potassium chloride, sodium nitroprusside (SNP), cromakalim and carbon monoxide releasing molecule (CORM) (Sigma-Aldrich). Stock solutions (10 mM) of cromakalim and CORM were prepared in absolute ethanol, and final ethanol concentration in the tissue baths never exceeded 0.1% (v/v). The remaining drugs were prepared in distilled water, except for NA which was dissolved in NaCl (0.9%)-ascorbic acid (0.01% w/v) solution. These solutions were kept at −20 °C and appropriate dilutions were made in KHS on the day of the experiment.

### Experimental animals and ethical procedures

Male Sprague-Dawley (SD) rats, 20–22 months old, were provided by the Animal Facility of the Universidad Autónoma de Madrid (UAM) (Registration number EX-021U). Systolic blood pressure was indirectly measured in awake animals by the tail-cuff method (Letica, Digital Pressure Meter, LE5000, Barcelona, Spain), and the animals were weighed before sacrifice. Rats were sacrificed by CO_2_ inhalation and subsequent decapitation; the aorta was carefully dissected out and placed in Krebs–Henseleit solution (KHS) at 4 °C containing: 115 mM NaCl, 2.5 mM CaCl_2_, 4.6 mM KCl, 1.2 mM KH_2_PO_4_, 1.2 mM MgSO_4_, 25 mM NaHCO_3_ and 11.1 mM glucose. The aorta was cleaned of adhering adipose and connective tissues, cut into rings of 4 mm in length, and divided into two groups: a control group (arteries incubated in KHS medium), and a *Spirulina* group (arteries exposed for 3 h to the aqueous *Spirulina* extract dissolved in the KHS medium [0.1% w/v]). All animal protocols were approved by the Research Ethics Committee of UAM according to directives 609/86 CEE and R.D. 233/88 of the Ministerio de Agricultura, Pesca y Alimentación of Spain (PROEX 202/16). The experiments were conducted in accordance with the published Guiding Principles in the Care and Use of Animals approved by the European Union directives 63/2010 UE and Spanish regulation RD53/2013.

### Aqueous extract of Spriulina

#### Biomass extraction

*Spirulina* was kindly supplied by ASN LEADER, S.L. (Murcia, Spain). An hydrophilic *Spirulina* extract was prepared by solvent extraction according to the Bligh and Dyer method (Bligh and Dyer 1959) after a prior step of *spirulina* biomass sonication. Briefly, 50 mg of *Spirulina* biomass suspended in 1 mL of chloroform/methanol 1:2 (v/v) was subjected to 40 kHz sonication for 15 min at 25 °C. The liquid phase was filtrated and the recovered biomass was extracted with 0.5 mL of chlorophorm/methanol 1:2 (v/v). Next, the hydrophilic *Spirulina* biocomponents were separated by adding twice 0.6 mL of water with 0.58% NaCl to the chloroform/methanol phase. After gravimetric separation of phases for 24 h at 4 °C, the aqueous phase was dried in a Buchi B480 rotary evaporator, weighed and frozen at −70 °C until use.

#### Elemental analyses

Lyophilised extract (70–80 mg) was digested in Teflon glasses with 6 mL HNO_3_ for 20 min in a Multiwave 3000 microwave ANTON PAAR model, with a program consisting on: starting from 0 to 500 W in 5 min, maintaining at 500 W for 10 min, and then increasing to 1000 W in 10 min, and maintaining at 1000 W for 20 min (maximal temperature program was set at 240 °C and maximal pressure value reached was 60 Bar). After digestion, the acid solution was diluted to 25 mL, and 0.5 mL of the corresponding solutions was diluted to 10 mL for the semiquantitative analysis. Sample was analysed by Inductive Coupled Plasma Mass Spectrometry (ICP-MS) in a NexION 300XX apparatus from PerkinElmer, with a method formerly described (Zuluaga et al. 2011).

#### Total carbohydrates content

Total carbohydrates content of lyophilised aqueous extract was determined by phenol-sulfuric method (DuBois et al. 1956). The extract was dissolved in milliQ water at a concentration of 0.2 g/L and analysed in triplicate, giving the results as the calculated mean value with their standard deviation.

#### Amino acid composition

Quantitative analysis of amino acids was carried out according with the procedure developed by Spackman et al. (1958) in a Biochrom 30 Series amino Acid Analyser, with a reproducibility >0.5 CV at 10 nmol Biochrom 30 uses the classic methodology for analysis of amino acids based in ion-exchange liquid chromatography and a post-column reaction in continuous with ninhydrin to obtain the qualitative and quantitative analysis, with a sensitivity of ∼10 pmol.

A solution of the dry aqueous extract (1–2.6 mg/mL) was prepared in triplicate in hydrolysis tubes, which contained a known concentration of norleucina (internal standard).

#### Peptide identification by LC ESI-MS MS

The aqueous extract was analysed by liquid chromatography coupled to an electrospray ionisation mass spectrometer in positive ionisation mode (LC/ESI-MS/MS), to identify the bio-components. Prior to analysis, the sample was cleaned with C18 tips, model ZipTip Pipette Tips C18 (ref. ZTC18S096 of Millipore). LC/ESI-MS/MS analyses were carried out in an Ultimate 3000 nanoHPLC (Dionex, Sunnyvale, CA, USA) coupled to an ion trap mass spectrometer AmaZon Speed (Bruker Daltonics, Bremen, Germany). The reversed phase analytic column used was an Acclaim C18 PepMap of 75 µm × 15 cm, 3 µm particle size and 100 Å pore size (ThermoScientific, USA). The trap column was a C18 PepMap of 5 µm particle diameter, 100 Å pore size, connected in series with the analytical column. The loading pump flushed a solution of 0.1% trifluoroacetic acid in 98% water/2% acetonitrile (ScharLab, Barcelona, Spain) at 3 µL/min. The nano-pump operated at a flow of 300 nL/min in gradient conditions, using 0.1% formic acid (Fluka, Buchs, Switzerland) in water (phase A), and 0.1% formic acid in 80% acetonitrile/20% water (phase B). The scheme of elution gradient was: isocratic mode with 96% A: 4% B for 5 min, a linear increase to 40% B in 60 min, a linear increase to 95% B in 1 min, isocratic conditions of 95% B for 7 min and return to initial conditions in 10 min. Five µL of extracts solutions (4 µg/µL) were injected, and detected at 214 and 280 nm wavelengths. In a second analysis 5 µL of extracts solutions (10 µg/µL) were injected. The LC system was connected by a CaptiveSpray source (Bruker Daltonics, Bremen, Germany) to the ion trap spectrometer, operating in positive mode with a capillary voltage set of 1400 V. The automatic data acquisition permitted to sequentially obtain both MS spectra (*m*/*z* 350–1500) and the MS CID spectra of the 8 more abundant ions. In the analyses of 10 µg/µL samples the MS spectra range was 100–1000 *m*/*z*. Exclusion dynamics was applied to prevent the isolation of the same *m*/*z* for 1 min after its fragmentation.

For peptide identification, MS and MS/MS data of individual fractions of HPLC were processed using DataAnalysis 4.1 (Bruker Daltonics, Bremen, Germany). MS/MS spectra (in form of generic Mascot files) were analysed against a data base obtained from NCBInr (National Centre for Biotechnology Information) containing 68623 entries of proteins from both *Spirulina* and *Arthrospira*. Database search was carried out with Mascot v.2.6.0 (Matrix Science, London, UK; Perkins et al. 1999). The search parameters were set as follows oxidised methionine as the modification variable without enzyme restriction. The tolerance for peptide mass was of 0.3 Da and 0.4 Da in MS and in MS/MS modes, respectively. In most of the cases, a precision of ±0.1–0.2 Da was obtained, both for MS and MS/MS spectra.

#### Total polyphenol content

The content of polyphenols and other antioxidants was determined by the Folin-Ciocalteu Reagent (FCR) method (Ainsworth and Gillespie 2007).

Prior to analyses, lyophilised aqueous extract was dissolved in 95% (v/v) methanol/water (10 g/L), and sonicated (20 kHz) for 10 min to get complete dissolution of the extract. Results are expressed as the mean value of data obtained in three replicas with the corresponding standard deviation (SD).

#### Antioxidant activity

For better characterisation of the antioxidant activity of the aqueous *Spirulina* extract two different methods were used:

##### ABTS assay

Mono-cation radical of 2,29-azinobis-(3-ethylbenzothiazoline-6-sulphonic acid) (ABTS•^+^) generated by oxidation of ABTS with potassium persulphate and it is reduced in presence of hydrogen donor antioxidants was determined (Re et al. 1999).

##### Hydroxyl radical scavenging assay (ORAC-Fluorescein assay)

Hydroxyl radical (OH^●^) is generated in living organism, having important negative effects in inflammatory processes of tissues of illness related with oxidative stress. The method described by Dávalos et al. (2004) was employed.

For ABTS and ORAC analyses, a multimodal plate reader SynergyTM HT with automatic dispenser of samples, and temperature control of Biotek Instruments (VT, USA) was used. The software Biotek Gen5TM was used for data analysis. Each plate with 96 wells was analysed in quadruplicate, with four standard levels of calibration and 8 repetitions for blank or control. Reaction was started by automatic addition of 60 μL of ABTS radical or AAPH to the sample solution for ABTS and ORAC assays, respectively. Antiradical activity with ABTS was determined after 10 min reaction. For ORAC method, the value was read after 180 min reaction. ABTS activity was determined in quadruplicate and ORAC activity in triplicate. The results were expressed by the TEAC (*Trolox equivalence antioxidant capacity*) value in mmol trolox/g extract, as the concentration (mM) of a standard reference solution (Trolox) with an antioxidant capacity equivalent to the one of a solution (1 mM) of the investigated analyte.

### Vascular reactivity

The method used for isometric tension recording has been described in full elsewhere (Nielsen and Owman 1971). Briefly, aortic segments were suspended in an organ bath containing 5 mL of KHS at 37 °C, continuously bubbled with 95% O_2_ and 5% CO_2_ mixtures (pH = 7.4). Two parallel stainless steel pins were introduced through the lumen of the vascular segment: one fixed to the bath wall and the other connected to a force transducer (Grass FTO3C; Grass Instruments Co., Quincy, MA, USA); this in turn was connected to a model 7D Grass polygraph. The aortic segments were subjected to a tension of 1 g which was adjusted every 15 min during a 90 min equilibration period before drug administration. Next, the vessels were exposed to 75 mM KCl to check the functional integrity. After a washout period the viability of vascular endothelium was tested by the ability of 10 µM ACh to relax precontracted segments with 0.1 µM NA. Vessels were then washed with KHS to recover the basal tension.

To investigate the effect of the *Spirulina* aqueous extract on the vasodilator response, separate aortic segments were incubated with the *Spirulina* extract (0.1% w/v) for 3 h (changes with a fresh *Spirulina* extract every 1 h) before performing cumulative concentration–response curves to ACh (0.1 nM–10 µM), to the NO donor sodium nitroprusside (SNP, 0.1 nM–10 µM), to the K_ATP_ channel opener cromakalim (CK, 0.1 nM–10 µM) and to the CO donor CORM (1 µM–100 µM) in 0.1 μM NA precontracted rings. The effect of *Spirulina* on the vasoconstrictor responses was also investigated by analysing the responses generated by KCl (75 mM) and by cumulative concentrations of NA (0.1 nM–10 µM).

### Release of nitric oxide (NO)

The release of NO was measured with a nitrite colorimetric assay kit (Cayman Chemical), as previously described (Villalpando et al. 2015). Briefly, during the last 30 min of the incubation period with either *Spirulina* or KHS the media (0.2 mL) was changed every 10 min, and the last incubation medium was collected to measure the basal NO release. Once the media were replaced, NA (0.1 µM, for 2 min) and ACh (10 µM for 8 min) were added and the medium was collected to measure the stimulated NO release. The media were stored at −80 °C until further analysis. The analysis was carried out according to the manufacturer’s protocol and the absorbance was measured at 540 nm. Also, blank measures were collected in the same way from medium without mesenteric segments to subtract background emission. Data were expressed as relative values compared to basal control condition.

### Detection of superoxide anion

Hydroethidine, an oxidative fluorescent probe, was used to evaluate superoxide anion levels *in situ*, as previously described (Martín et al. 2005; Sagredo et al. 2013; Villalpando et al. 2015). The tissue was also stained with the nuclear dye 4′,6-diamidino-2-phenylindole (DAPI, 10 μg/mL). Segments were mounted on glass slides and imaged on a confocal microscope. Images were obtained with a LEICA (TCS ST2 DM IRE2) laser scanning confocal microscope to detect nuclei (405 nm excitation and 410–475 nm emission, for the DAPI dye) and oxidised HE (excitation 488 nm, emission 610 nm). Laser and image settings were unchanged for the acquisition of images from the three groups of rats. The photomicrographs show the intensity and location of HE, which reflects superoxide production, so that comparison of these groups could be made. To analyse fluorescence intensity the ImageJ Analysis Software (National Institutes of Health [NIH]) was used. The amount of superoxide formation was expressed as the ratio between the fluorescence emitted by HE and that emitted by DAPI.

### Western blot analysis of p-eNOS and HO-1 expression

Arterial segments were homogenised and processed to quantify protein concentration at 4 °C in RIPA buffer containing phosphatase inhibitors and a cocktail of protease inhibitors. Proteins (20 µg) were separated by SDS-PAGE gels, and transferred to polyvinylidene difluoride (PVDF) membranes (Bio Rad Immun-Blot^®^ overnight at 4 °C, 230 mA, using a Bio-Rad Mini Protean III system (Bio-Rad Laboratories, Hercules, CA, USA). Membranes were blocked with 5% (w/v) fat-free powdered milk or 5% (w/v) bovine serum albumin following the instructions of the antibodies’ manufactures, and incubated overnight with mouse monoclonal antibody for eNOS (1:250 dilution), purchased from Transduction Laboratories (Lexington, UK), or with rabbit polyclonal antibody for HO-1 (1:2000 dilution), purchased from Stresggen Bioreagents (Victoria, Canada). After washing, the membrane was incubated with the corresponding anti-Immunoglobulin G conjugated to horseradish peroxidase (Amersham International Plc). The membrane was thoroughly washed and the immunocomplexes were detected using an enhanced horseradish peroxidase/luminol chemiluminiscence system (ECL Plus, Amersham International Plc, Little Chalfont, UK) and subjected to autoradiography (Hyperfilm ECL, Amersham International Plc). Signals on the immunoblot were quantified using a computer program (NIH Image V1.56). The same membrane used for eNOS detection was subjected to stripping and processed with the mouse monoclonal antibody for phospho-eNOS (1:500 dilution). Sections of the same membranes were used to determine GAPDH expression as loading control, by means of a monoclonal antibody anti GAPDH (1:5000 dilution, Sigma). The phosphorylation of eNOS was expressed as the p-eNOS/eNOS signals ratio. The expression of HO-1 was indicated as HO-1/GAPDH signals ratio.

### Data analysis

Results are given as means ± SEM (Standard Error of the Mean). The relaxation induced by ACh, SNP, cromakalim and CORM was expressed as a percentage of initial contraction elicited by NA. The vasoconstrictor response induced by KCl (75 mM) and that induced by NA were expressed in mg. Statistical analysis was performed by comparing the curves obtained in aortae from SD rats after *Spirulina* extract incubation with that obtained in the absence of the extract by means of two-way analysis of variance (ANOVA). The maximum response (*E*_max_ values) and the logarithm of the concentration of ACh, SNP, CORM, cromakalim and NA producing 50% of maximum response (log EC_50_) were calculated by a nonlinear regression analysis of each individual concentration–response curve using Graph Pad Prism Software (San Diego, CA). For *E*_max_, log EC_50_, NO release, superoxide anion production and proteins expression, statistical analysis was done using Student’s *t*-test for unpaired experiments. A *p* value of less than 0.05 was considered significant. The statistical analysis and the elaboration of the graphs were carried out using the statistical program GraphPad PRISM^®^ (Version 6.01).

## Results

### Composition and antioxidant activity of the aqueous Spirulina extract

The aqueous *Spirulina* extract was obtained with 19.20 ± 0.20% weight yield. The elemental composition analysis of extract indicated that it was essentially free of toxic elements (0 ppm Hg; 0.1 ppm Cd; 3.0 ppm As; 2.9 ppm Ni), and had variable amounts of other elements including some essential ones (33037 ppm K; 3177 ppm Mg; 11.7 ppm Mn; 1576 ppm Ca; 2.7 ppm Cu; 4.2 ppm Fe; 0 ppm Se; 0 ppm Zn).

Total carbohydrate content of the extract was of 336 ± 44 mg/g extract.

This lyophilised extract had relatively high amino acid content (34% w/w) and contained all types of amino acids, particularly the essential ones (Table 1). Twelve peptides were identified in the lyophilised extract (Table 2). Part of them corresponded to fragments of chlorophyll binding protein and phycocyanin (responsible for photosynthetic and antioxidant activities). Others were fragments of phosphoenolpyruvate synthase. Analyses using *Spirulina* and *Arthrospira platensis* data bases revealed that in the extract there is a significant presence of peptides (Table 2).

**Table 1.** Amino acid content of the lyophilised *Spirulina* aqueous extract.

Amino acid	% w/w	µmol/g extract
Asp	3.30 ± 0.04	287.17 ± 3.59
Thr	1.86 ± 0.02	161.54 ± 1.45
Ser	1.93 ± 0.02	167.83 ± 1.81
Glu	7.16 ± 0.11	622.12 ± 9.17
Pro	1.57 ± 0.02	136.36 ± 1.73
Gly	1.54 ± 0.02	134.09 ± 2.15
Ala	3.46 ± 0.05	300.51 ± 4.72
Cys	0.31 ± 0.00	26.70 ± 0.14
Val	1.79 ± 0.04	155.32 ± 3.13
Met	0.74 ± 0.01	64.64 ± 1.06
Ile	1.56 ± 0.03	135.79 ± 2.45
Leu	2.91 ± 0.07	252.66 ± 5.65
Tyr	0.70 ± 0.01	60.62 ± 1.02
Phe	1.81 ± 0.04	157.16 ± 3.14
His	0.38 ± 0.01	33,29 ± 0.58
Lys	1.68 ± 0.03	145.63 ± 3.03
Arg	1.66 ± 0.02	144.40 ± 2.17
Total	34.36 ± 0.54	2985.83 ± 46.98

**Table 2.** Peptide sequence, retention time (Rt, min), calculated and experimental molecular mass (Da), and mark or score of the identified peptides in the aqueous extract of *Spriulina* by LC ESI-MS MS.

RT (min)	Peptide sequence (Seq ID)	*M*_r_ (Da, Calc.)	*M*_exp_ (Da)	Mark	Data base
42.46	NGDPFVGHL (SEQ ID NO: 83)	954.46	954.46	43	gi|291569436, photosystem I reaction centre subunit XI [*Arthrospira platensis* NIES-39]
49.82	VFETGIKVVDL (SEQ ID NO: 84)	1218.69	1218.64	53	gi|291568724, ATP synthase beta chain [*Arthrospira platensis* NIES-39]
49.83	DFFVDKL (SEQ ID NO: 85)	882.45	882.43	42	gi|291571801, phosphoenolpyruvate synthase [*Arthrospira platensis* NIES-39]
50.10	GPPLDIKL (SEQ ID NO: 86)	851.51	851.48	37	gi|291565679, iron-stress induced chlorophyll-binding protein [*Arthrospira platensis* NIES-39]
50.50	DVNETVLDNLPKTRTQI (SEQ ID NO: 87)	1955.03	1954.97	41	gi|209495148, phosphoenolpyruvate synthase [*Arthrospira maxima* CS-328]
53.41	DVNETVLDNLP (SEQ ID NO: 88)	1227.6	1227.5	73	gi|209495148, phosphoenolpyruvate synthase [*Arthrospira maxima* CS-328]
56.10	DSLISGAAQAVY (SEQ ID NO: 89)	1193.59	1193.59	37	gi|10302997, phycocyanin alpha subunit, partial [*Arthrospira sp*. Paracas P2]
57.09	GIGNDPLEIQF (SEQ ID NO: 90)	1201.6	1201.6	57	gi|291565650, phycobilisome core-membrane linker polypeptide [*Arthrospira platensis* NIES-39]
57.76	GLILLPHLATL (SEQ ID NO: 91)	1159.73	1159.62	46	gi|495331734, chlorophyll a/b binding light-harvesting protein [*Arthrospira sp*. PCC 8005]
58.80	GLILLPHLA (SEQ ID NO: 92)	945.6	945.6	39	gi|291565679, iron-stress induced chlorophyll-binding protein [*Arthrospira platensis* NIES-39]
58.90	AVLGAGALFHTF (SEQ ID NO: 93)	1202.64	1202.68	45	gi|291565679, iron-stress induced chlorophyll-binding protein [*Arthrospira platensis* NIES-39]
60.60	DVNETVLDNLP (SEQ ID NO: 94)	1227.6	1227.6	59	gi|209495148, phosphoenolpyruvate synthase [*Arthrospira maxima* CS-328]

The polyphenol content of this *Spirulina* extract determined by the FCR method was of 9.59 ± 0.03 mg/g extract. The *Spirulina* extract exhibited antioxidant activities determined by ABTS and ORAC methods of 23.4 ± 0.9 and 199 ± 16 TEAC (µmol Trolox/g extract), respectively.

### Animal weight and systolic blood pressure

The body weight (709.4 ± 10.93 g) and the systolic blood pressure values of the aged rats (162 ± 4.7mmHg) were similar to those reported in previous studies (Ferrer and Balfagón 2001; Ferrer et al. 2003).

### Vascular reactivity

**Figure 1.**
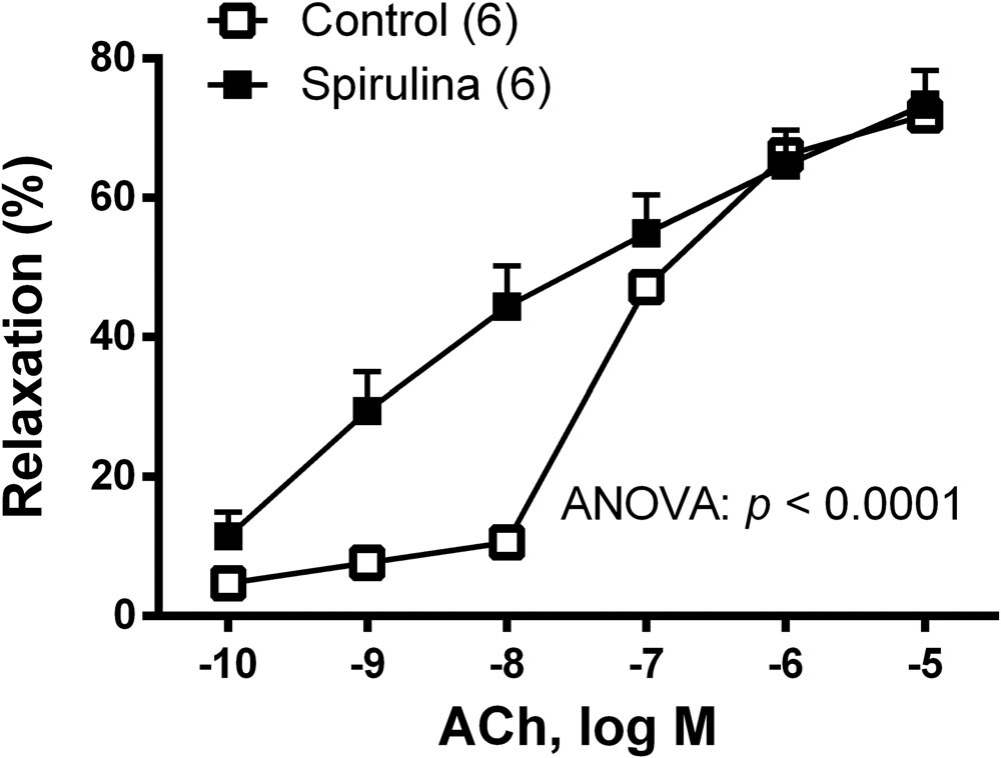
Effect of the aqueous *Spirulina* extract incubation on the concentration–response curve to acetylcholine in aortic segments from aged SD rats. Results (means ± SEM) are represented as the percentage of inhibition of the contraction elicited by 0.1 µM noradrenaline. Number of animals is indicated in parenthesis. The statistical significances are indicated in the corresponding graphs.

In NA-precontracted arterial segments, the vasodilator response induced by ACh (0.1 nM–10 µM) was increased after *Spirulina* extract incubation (Figure 1).

**Figure 2.**
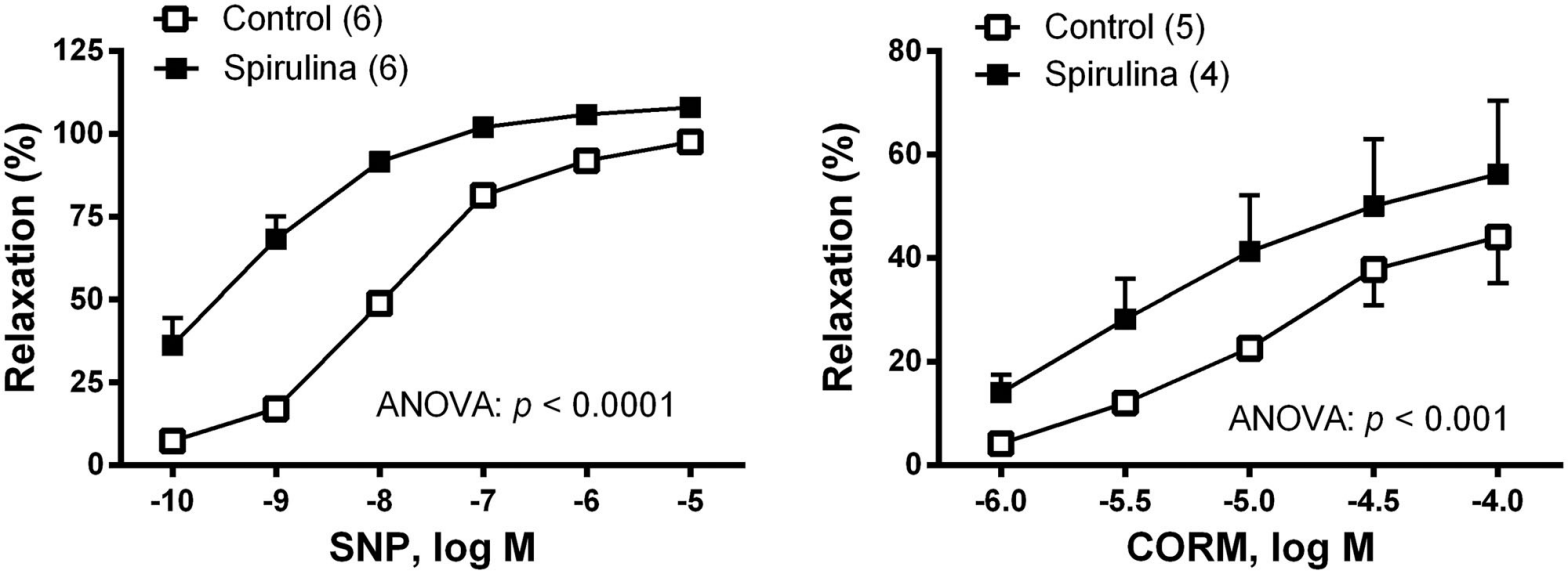
Effect of the aqueous *Spirulina* extract incubation on the concentration–response curve to the NO donor sodium nitroprusside (SNP), and to the CO releasing molecule (CORM), in aortic segments from aged SD rats. Results (means ± SEM) are presented as percentage of inhibition of the contraction elicited by 0.1 µM noradrenaline. Number of animals is indicated in parenthesis. The statistical significances are indicated in the graph.

To analyse the possible action of *Spirulina* on the sensitivity of smooth muscle cells to NO, the vasodilator response induced by the NO donor, SNP was studied. After incubation with *Spirulina* extract, the vasodilator response elicited by SNP (0.1 nM–10 µM) was increased (Figure 2). To analyse the possible action of *Spirulina* on the sensitivity of smooth muscle cells to CO, the vasodilator response induced by the CO donor, CORM (1 µM–100 µM) was studied. After incubation with *Spirulina* extract, the vasodilation response induced by CORM was increased (Figure 2).

**Figure 3.**
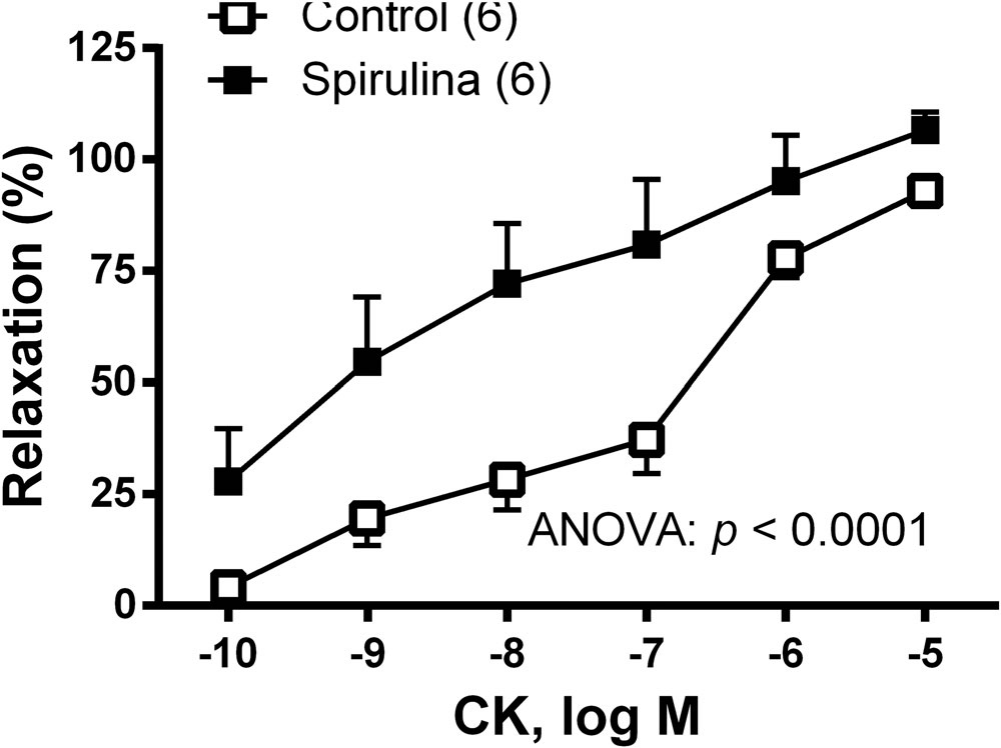
Effect of the aqueous *Spirulina* extract incubation on the concentration–response curve to the K_ATP_ channel opener cromakalim (CK) in aortic segments from aged SD rats. Results (means ± SEM) are represented as percentage of inhibition of the contraction elicited by 0.1 µM noradrenaline. Number of animals is indicated in parenthesis. The statistical significances are indicated in the graph.

To analyse the possible action of *Spirulina* extract on the function of K_ATP_ channels, concentration–response curve to the K_ATP_ channels opener cromakalim (0.1 nM–10 µM) was performed. The results showed that the vasodilator response to cromakalim was enhanced after incubation with *Spirulina* extract (Figure 3).

**Figure 4.**
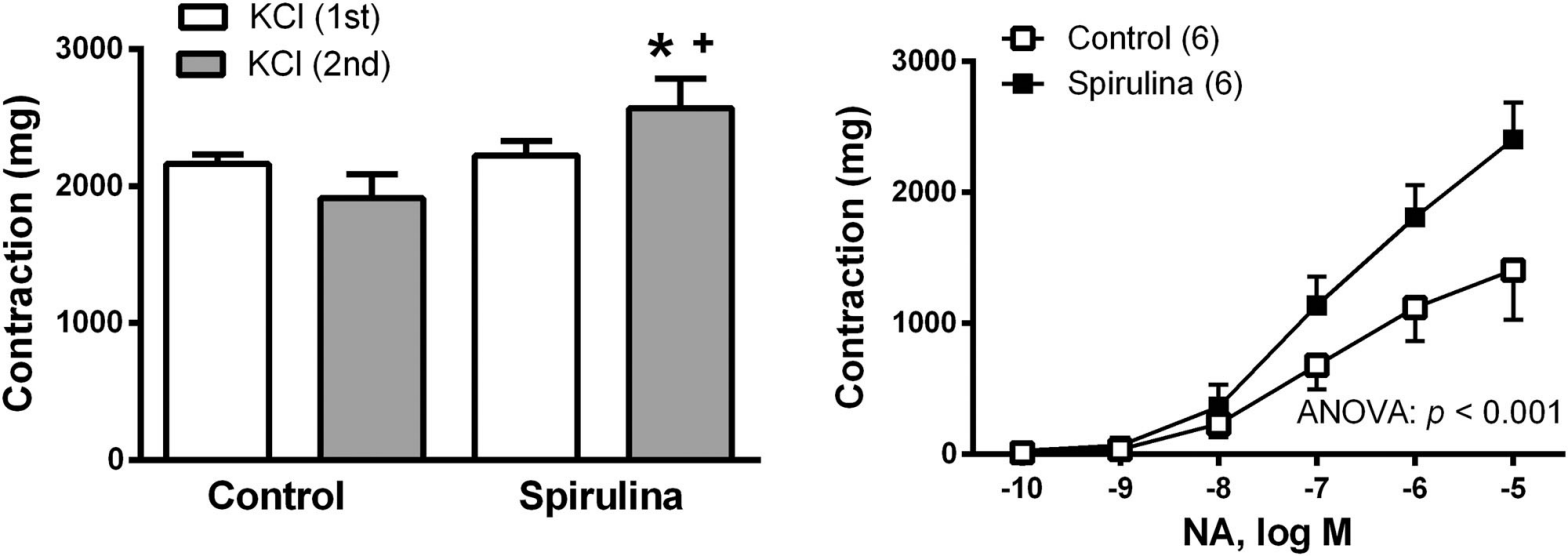
Effect of the aqueous *Spirulina* extract incubation on the response induced by 75 mM KCl and the concentration–response curve to noradrenaline (NA) in aortic segments from aged SD rats. Results (means ± SEM) are represented as mg of contraction. Number of animals is indicated in parenthesis. The statistical ANOVA significance is indicated in the graph. *p < 0.05 compared with respective basal release; ^+^*p* < 0.05 compared with control condition (in the absence of *Spirulina* extract).

The vasoconstrictor response elicited by 75 mM KCl was increased by 1.3-fold (*p* = 0.0285) after incubating the vessels with *Spirulina* extract (in mg: control: 1912 ± 176.1; *Spirulina*: 2569 ± 217.1; Figure 4). The vasoconstrictor response induced by NA (0.1 nM–10µM) was also increased by 1.7-fold (*p* = 0.045) after incubation with the aqueous *Spirulina* extract (in mg: control: 1407 ± 379.5; *Spirulina*: 2403 ± 283.4; Figure 4).

The effect of the aqueous *spirulina* incubation on the values of *E*_max_ and the pEC_50_ elicited by ACh, SNP, CORM, CK and NA were included in Table 3.

**Table 3.** Effect of the aqueous *Spirulina* extract incubation on the maximal vasomotor response (*E*_max_) and on the logarithm of effective concentration 50 (pEC_50_) of acetylcholine (ACh), sodium nitroprusside (SNP), CO releasing molecule (CORM), cromakalim (CK) and noradrenaline (NA) in aorta from aged rats.

	*E*_max_		pEC_50_	
	Control	*Spirulina*	Control	*Siprulina*
*ACh*	71.82 ± 5.47	73.36 ± 4.93	–7.06 ± 0.11	–8.16 ± 0.21**
*SNP*	97.50 ± 1.67	108.0 ± 2.08**	–7.96 ± 0.16	–9.11 ± 0.14**
*CORM*	44.0 ± 8.8	56.25 ± 14	–4.99 ± 0.13	–5.23 ± 0.07
*CK*	93 ± 3.07	106.7 ± 3.98*	–7.05 ± 0.39	–8.29 ± 0.53*
*NA*	1407 ± 379	2403 ± 283*	–6.89 ± 0.20	–6.76 ± 0.16

Values (mean ± SEM) are expressed as a percentage of relaxation (ACh, SNP, CORM and CK) and in mg (NA) for *E*_max_ and as log EC_50_ for pEC_50_. Statistical significances: *p < 0.05, **p < 0.001 compared to control condition (in the absence of the *Spirulina* extract).

### Nitric oxide release

**Figure 5.**
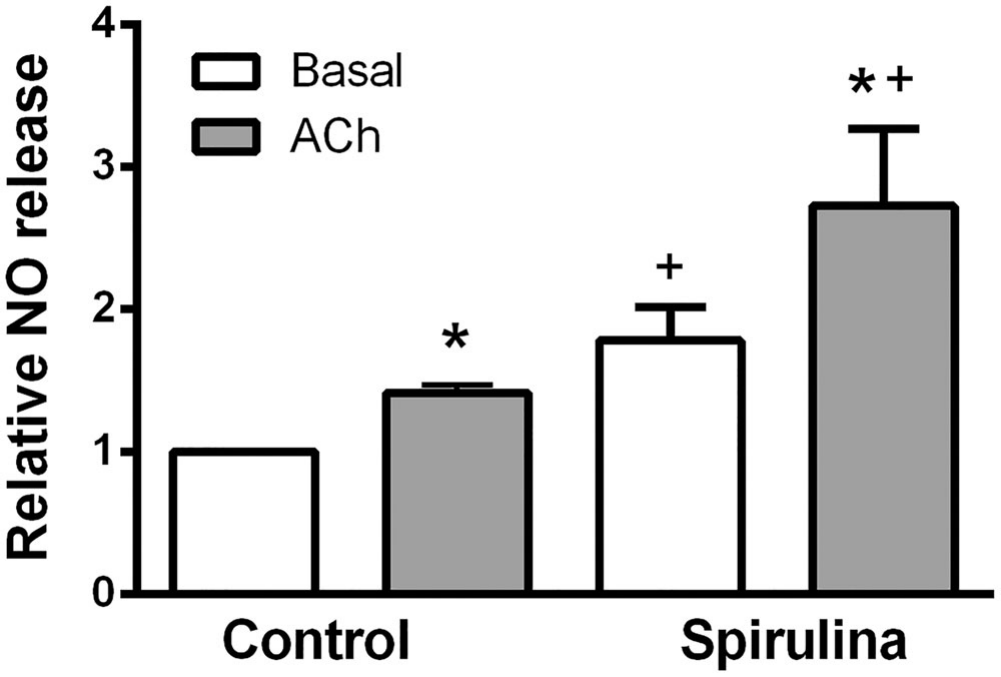
Effect of the aqueous *Spirulina* extract incubation on the basal and ACh-induced NO release in aortic segments from aged SD rats. Results (means ± SEM) are expressed relative to the basal release in control condition (=1). Number of animals: 5–7. *p < 0.05 compared with respective basal release; ^+^*p* < 0.05 compared with control condition (in the absence of *Spirulina* extract).

The nitrite content under basal and ACh-stimulated conditions was increased by 1.4-fold (*p* = 0.0022) and by 1.9-fold (*p* = 0.0087), respectively in arteries exposed to the aqueous extract of *Spirulina* with respect to the control condition (basal: control: 1.0 ± 0.0; *Spirulina*: 1.78 ± 0.231 and ACh-stimulated: control: 1.415 ± 0.054; *Spirulina*: 2.725 ± 0.544). ACh exposure increased the nitrite content more in arteries incubated with *Spirulina* (1.5-fold) than in control ones (1.4-fold; Figure 5).

### Detection of superoxide anion

**Figure 6.**
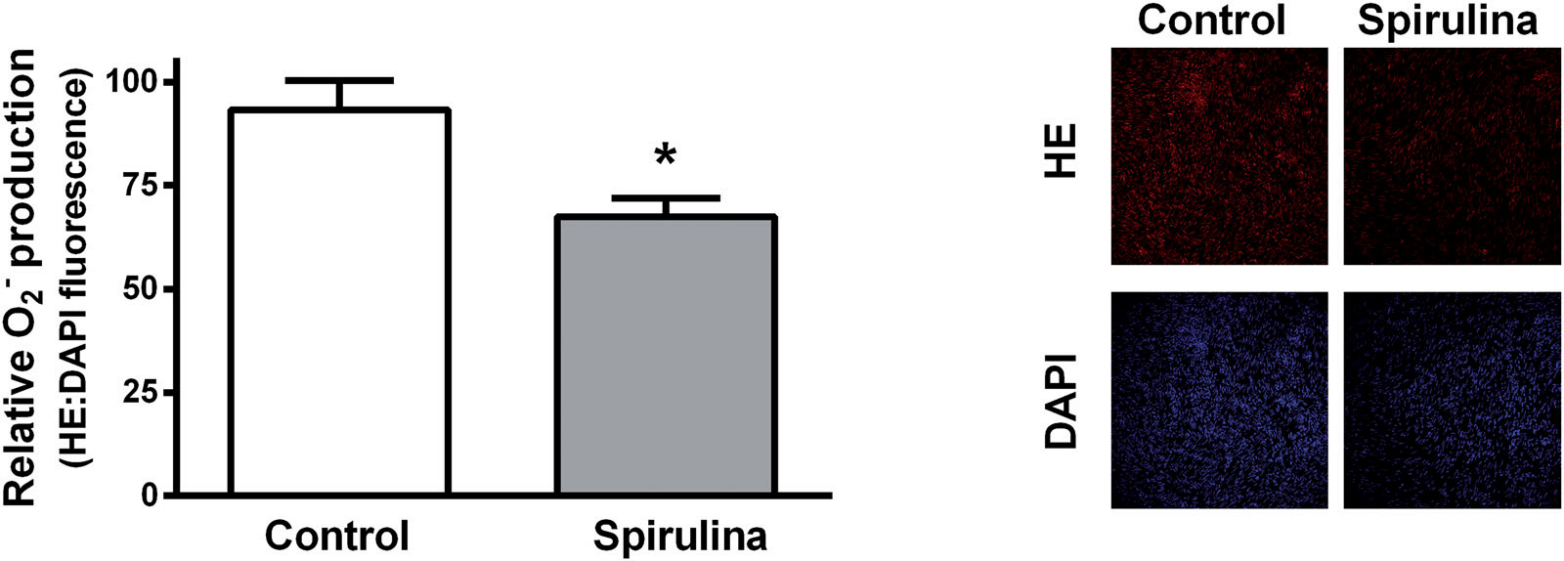
Effect of the aqueous *Spirulina* extract incubation on the production of superoxide anion in aortic segments from aged SD rats. Representative confocal images showing *in situ* detection of superoxide anion in red, and DAPI-nuclei staining in blue. Quantitative analysis of fluorescence is also shown. Results (means ± SEM) are expressed as the ratio between the fluorescence emitted by HE and that emitted by DAPI. Number of animals: 4.

In arteries from aged SD rats, the incubation with *Spirulina* extract significantly reduced by 0.7-fold (*p* = 0.007) the production of superoxide anion indicated by the diminished HE fluorescence (control: 93.19 ± 7.15; *Spirulina*: 67.39 ± 4.55). The *Spirulina* extract did not modify the fluorescence emitted by DAPI (Figure 6).

### Expression of p-eNOS and HO-1

**Figure 7.**
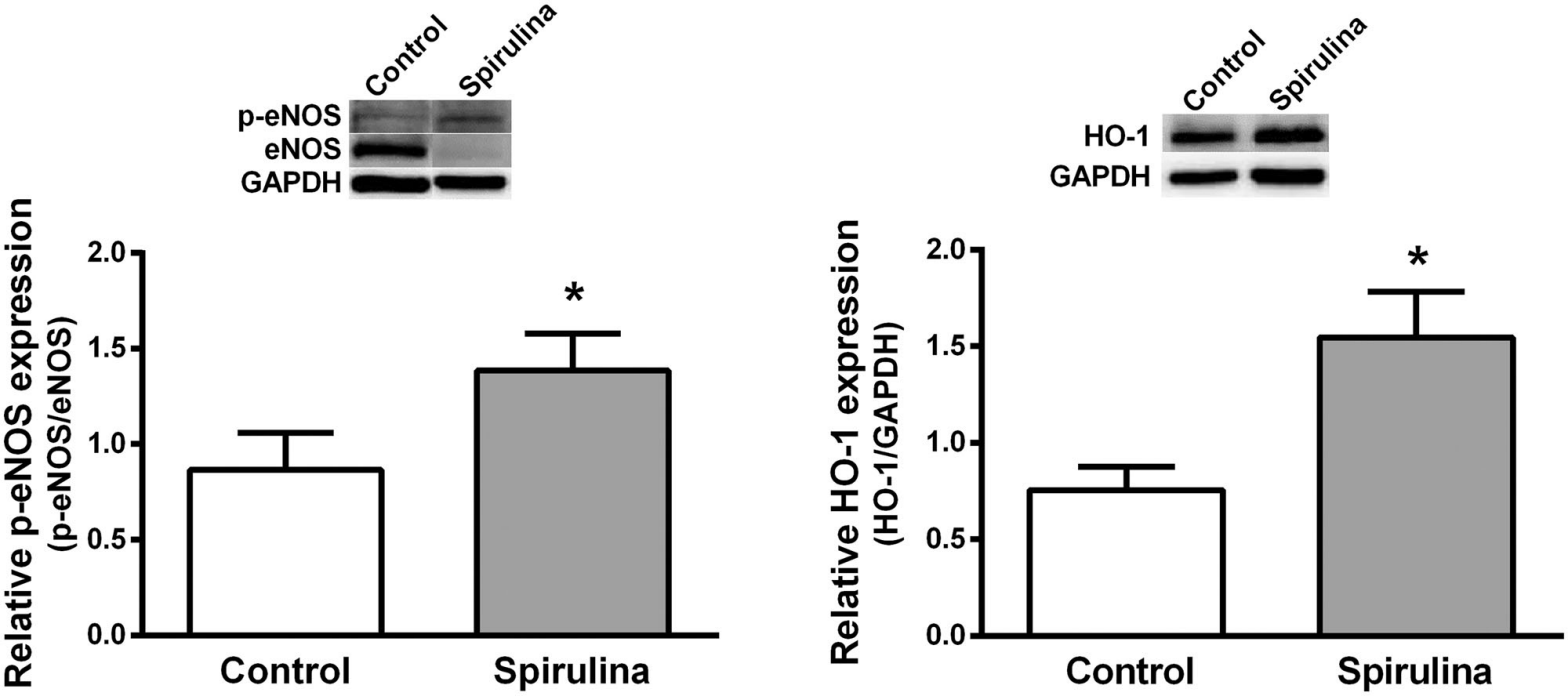
Representative western blot and densitometric analysis for the expression of p-eNOS and HO-1 protein in aortic segments from aged SD rats in the absence (control) or in the presence of the aqueous *Spirulina* extract. Results (means ± SEM) are expressed as peNOS/eNOS or HO-1/GAPDH signals ratio. Number of animals: 4–6. *p < 0.03 compared with control condition (in the absence of *Spirulina* extract).

The effect of *Spirulina* extract on the expression of p-eNOS and HO-1 was analysed in homogenates of aortae from old SD rats by using western blot analysis. The results show that the expression of p-eNOS and HO-1 were increased after *Spirulina* incubation by 1.6-fold (control: 0.865 ± 0.196; *Spirulina*: 1.386 ± 0.192, *p* = 0.045) and by 2.0-fold (control: 0.755 ± 0.120; *Spirulina*: 1.545 ± 0.238, *p* = 0.0143), respectively (Figure 7).

## Discussion

Overall, the present work shows that incubation with the aqueous extract of *Spirulina* improves the vasomotor response in the aorta of aged rats. Incubation for 3 h with the extract increased the release of NO and decreased the production of superoxide, effects which was linked to the increased expression of p-eNOS and HO-1. In addition, the increase in the vasodilator action induced by NO, CO and by K_ATP_ channel activation may account for the increased ACh-induced relaxation observed after incubation with the *Spirulina* extract. The vasoconstrictor response induced by KCl and NA was also improved. The antioxidant activity of the *Spirulina* extract could contribute, at least in part, to the observed functional effects.

The physiological process of ageing leads to a gradual decline in functionality of the different systems that form the whole organism. In relation to the vascular system, hallmarks of ageing include endothelial dysfunction and vascular stiffness, which are associated with decreased NO bioavailability and increased ROS production (Brandes et al. 2005; Wu et al. 2014; Thijssen et al. 2016; Ungvari et al. 2018). Recently, the consumption of natural products with antioxidant properties (such as microalgae extracts) has increased (Vo and Kim 2013; Heo et al. 2017). We have previously reported that incubation with the aqueous *Spirulina* extract improved the vasodilator response of the aorta from hypertensive rats through increased NO release/function (Villalpando et al. 2020). Therefore, we start by analysing the endothelium-dependent relaxation. The results showed that the ACh-induced relaxation was increased in *Spirulina* incubated vessels from aged rats, as previously observed in vessels of hypertensive rats (Villalpando et al. 2020). Due to this result, the effect of *Spirulina* incubation on the production of endothelial NO was studied. The results demonstrated that the incubation with the *Spirulina* extract increased the basal and the ACh-induced NO release, as reported in previous studies (Carrizzo et al. 2019; De Freitas Brito et al. 2019; Villalpando et al. 2020). In the aorta of aged rats, the increase in the NO production seems to be linked to the increase of p-eNOS as demonstrated by western blot experiments, although an increase in NO bioavailability cannot been ruled out. Therefore, and based on the antioxidant properties described for *Spirulina* (Khan et al. 2005; Castro-García et al. 2018), the effect of spirulina on the content of superoxide anion was investigated. The results clearly demonstrated the reduction of superoxide anion after incubation with the *Spirulina* extract which matches previous studies (Ali et al. 2015; Wu et al. 2016; Rajasekar et al. 2019). Beyond this effect, what it is important to note is that HO-1 is one of the earliest expressed proteins in response to a pro-oxidant environment, as occurs in ageing, and that exerts protecting vascular functions (Kang et al. 2015; Drummond et al. 2019). Keeping this information in mind, the possible influence of *Spirulina* extract on the expression of this protein was studied. The results demonstrated that HO-1 expression was enhanced after incubating the vessels with *Spirulina*, which is in agreement with the antioxidant properties of the *Spirulina* described in glial cells (Morita et al. 2015) and in the aorta of hypertensive rats (Villalpando et al. 2020). Thus, the use of natural antioxidant compounds is currently a very active area of research, since it could have an impact on different diseases that match with chronic inflammation (e.g., cancer, neurodegenerative and vascular diseases). In fact, activation of Nrf2 and HO-1 has been described as a central event underlaying the antioxidants properties of an important number of natural compounds (Howden 2013; Kang et al. 2015; Drummond et al. 2019). Additionally, different studies have shown that many compounds can display hormetic responses, i.e., biphasic responses depending on its concentration, the targeted cell type and even the temporal response profile (Calabrese et al. 2010; Dattilo et al. 2015; Calabrese 2018; Jodynis-Liebert and Kujawska 2020). The study of the concept of hormesis deserves future research that will allow a better understanding of their impact on different pathologies treatment.

The results discussed so far show that the aqueous *Spirulina* extract enhances the endothelium-dependent relaxation through increased NO release and bioavailability. Taking into account that increased oxidative stress, which occurs in vascular ageing, may reduce the effectiveness of the NO/cGMP/cGK axis (Chen et al. 2000; Lin et al. 2001), the analysis of the influence of *Spirulina* extract on the smooth muscle sensitivity to NO is essential. The results showed that the SNP-induced relaxation was increased by the extract which is in line with studies describing an increased NO involvement in the responses induced by different compounds after *Spirulina* treatment (De Freitas Brito et al. 2018; Carrizzo et al. 2019). Similarly, to what was found in the aorta from hypertensive rats (Villalpando et al. 2020), the greatest increase in the SNP-induced relaxation after *Spirulina* incubation was observed at low concentrations of SNP indicating the physiological relevance of that effect.

Since CO -synthesized from HO-1 by the degradation of haem to biliverdin/bilirubin- is able to induce relaxation (Leffler et al. 2006; Ryter et al. 2006), the influence of *Spirulina* on the sensitivity of the smooth muscle to the CO was also studied. The results showed that the relaxation induced by CORM was enhanced by *Spirulina*. To our knowledge, this is the first report describing *Spirulina* actions on the relaxation induced by CO. NO and CO share similar mechanisms of action because both gas mediators are able to hyperpolarize the cell membrane by activating K_ATP_ channels (Félétou and Vanhoutte 1996; Hussain et al. 1997; Foresti et al. 2004). Since a decrease in K_ATP_ channels functionality has been described in ageing (Ferrer et al. 1998; Liu et al. 2021), the effect of *Spirulina* extract on the function of K_ATP_ channels was analysed. The results showed that the vasodilator response induced by cromakalim, a K_ATP_ channel opener, was increased in vessels incubated with *Spirulina*, which could indicate an increased participation of hyperpolarizing mechanisms. This finding is in line with what was reported in the aorta of hypertensive rats (Villalpando et al. 2020) and future research is required to describe how the *Spirulina* extract achieves this specific effect.

During ageing, arteries became less responsive to both vasodilator and vasoconstrictor agents (Docherty 1988; Hashimoto et al. 1998). It has been reported that changes in calcium signalling underlying decreased vascular contractility may include alterations in: (i) the calcium entry to the cell, (ii) the calcium release from intracellular stores to the cytosol, (iii) the activity of calcium-ATPases involved in the maintenance of low cytoplasmic calcium concentration, and (iv) the structural changes of caveolae which are critical for vascular function (Ermak and Davies 2002; Fan et al. 2020; Harraz and Jensen 2020).

Despite these possibilities, we investigated the effect of the *Spirulina* extract on the responses elicited by KCl and by NA, as examples of two different mechanism of action that induce contraction. It is widely recognised that a high KCl concentration induces membrane depolarisation which stimulates calcium entry through L-type voltage operated calcium channels (L-VOCCs), while NA works by activating α-adrenoceptors inducing the breakdown of phosphatidylinositol-4,5-bisphosphate to inositol 1,4,5-triphosphate (IP_3_) and 1,2-diacylglycerol (DAG; Khalil and van Breemen 1988). IP_3_ stimulates calcium release from intracellular stores (Berridge and Irvine 1984; Suematsu et al. 1984), meanwhile DAG activates protein kinase C (Nishizuka 1992).

Our results demonstrated that the responses induced by KCl and NA were increased after *Spirulina* incubation. These results are in line with those describing an increase in the contractile response in aortic rings of hypertensive rats (Arthur-Ataam et al. 2019) and of hamsters on a high fat diet (Vidé et al. 2015) after supplementation with silicon-enriched *Spirulina* after 3 months. The authors suggest that silicon-enriched *Spirulina* is able to prevent the extracellular matrix degenerative processes that occur in the used pathological animal models, i.e., hypertension and in atherosclerosis. However, in our experimental conditions, where the incubation period is 3 h, this possibility is ruled out; however, it could be possible that the *Spirulina* extract, by reducing different ROS, could contribute to improving the contractile responses. In this respect, increased oxidative stress has been related to impairment of contraction in ageing through modulating calcium handling (Rueckschloss et al. 2010; Trebak et al. 2010; Andersson et al. 2011; Vidé et al. 2015) and myofilament function (Huveneers et al. 2015; Lacolley et al. 2017; Xu et al. 2017). Considering this information, together with the results described in the current study, future research on the detailed molecular mechanisms underlying the increased contractility induced by *Spirulina* is warranted, as already indicated for the eNOS/NO/sGC/cGMP axis (Münzel and Daiber 2019).

Overall, the results presented in this study, as well as considering the beneficial effects of *Spirulina* extract, open a series of future investigations that will serve to further understand the molecular mechanisms by which *Spirulina* influences vascular functioning. In addition, an interesting research field could consist of analysing the vasculoprotective effects of different concentrations of the *Spirulina* extract. This would be of great relevance to transfer these results to *in vivo* studies to establish a low-concentration hormetic protection, as has already been suggested (Calabrese 2018; Calabrese et al. 2000, 2007, 2010; Dattilo et al. 2015). Future investigation to determine the optimal hormetic-response induced by different compounds activating mechanisms able to counteract pathological events could be of great interest, as already demonstrated for phytochemicals (Jodynis-Liebert and Kujawska 2020). Related to the *in vivo* experiments, it would be desirable to perform screening of potential toxic compounds causing side effects. In this sense, and although human consumption of food supplements is approved by food regulatory agencies, the content of different toxins in microalgal products has been reported (Rzymski et al. 2019; Henao et al. 2020). Additional, individual characteristics of the consumers (allergies, hormonal status, among others) should be considering before initiating consumption of the supplement.

## Conclusions

The current study demonstrates that incubation with an aqueous *Spirulina* extract improves the vasomotor function of the aorta from aged rats. The increased expression of p-eNOS and HO-1 seems to be linked to the increased release of NO and to the decreased production of superoxide. Also, the increase in the vasodilator action induced by NO, CO and by K_ATP_ channel activation appears to be involved in the increased ACh-induced relaxation observed after incubation with the *Spirulina* extract. Likewise, the antioxidant properties of *Spirulina* could be mediating the improvement of the contractile responses.

## Data Availability

The data used to support the findings of this study are included within the article.
